# NDUFA4 Deletion Upregulates VDAC1 to Promote Mitochondrial Damage, Endoplasmic Reticulum Expansion, and Neuronal Apoptosis

**DOI:** 10.1155/humu/8889386

**Published:** 2026-05-21

**Authors:** Lushan Li, Fang Fu, Ru Li, Hang Zhou, Ruibing Huang, Jianqin Lu, Fei Guo, Huanyi Chen, Tingying Lei, Jin Han, Li Zhen, Min Pan, Dongzhi Li, Can Liao

**Affiliations:** ^1^ Department of Prenatal Diagnostic Center, Guangzhou Women and Children′s Medical Center, Guangzhou Medical University, Guangzhou, Guangdong, China, gzhmc.edu.cn

**Keywords:** Dandy–Walker malformation, ER, mitochondrial damage, NDUFA4, VDAC1

## Abstract

**Background:**

Dandy–Walker malformation (DWM) is a rare congenital brain defect whose mechanism is still not fully understood. Genetic studies have suggested that NADH Dehydrogenase 1 alpha Subcomplex 4 (NDUFA4) may be associated with DWM; however, the functional consequences of NDUFA4 dysregulation in experimental neural models remain unclear. In this study, we investigated the cellular effects of NDUFA4 deficiency, rather than aiming to establish a causal mechanism for DWM.

**Methods:**

NDUFA4‐related differentially expressed proteins were analyzed using iTRAQ tandem mass spectrometry, followed by functional, pathway, and protein interaction network analyses. Then, voltage‐dependent anion Channel 1 (VDAC1), apoptosis‐, endoplasmic reticulum (ER) stress–related proteins, and ETC Complex IV activity were assessed in NDUFA4 knockout mice. After NDUFA4 and VDAC1 knockdown, cell proliferation, apoptosis, mitochondrial status, and ER function were evaluated by CCK‐8, Edu staining, flow cytometry, and Western blot in C8‐D1A cells. In addition, the interaction between NDUFA4 and VDAC1 was evaluated using immunofluorescence and coimmunoprecipitation.

**Results:**

NDUFA4 knockout upregulated VDAC1, ER stress‐ and apoptosis‐related proteins, and inhibited ETC complex IV activity in mice. NDUFA4 knockdown inhibited proliferation and promoted apoptosis, ER stress, and mitochondrial damage in C8‐D1A cells, and these changes were partially reversed by VDAC1 knockdown. Moreover, NDUFA4 and VDAC1 colocalized in C8‐D1A cells and mouse cerebellar tissue, and NDUFA4 was found to interact with VDAC1.

**Conclusions:**

NDUFA4 deletion was associated with VDAC1 upregulation, mitochondrial damage, ER stress, and apoptosis‐related changes in our experimental models. These findings may provide insight into cellular changes potentially relevant to DWM; however, they do not establish a direct causal relationship.

**Highlights**

1. NADH Dehydrogenase 1 alpha Subcomplex 4 (NDUFA4) knockout upregulated VDAC1 expression, activated ER stress, and promoted apoptosis.
2. Knockdown of NDUFA4 promoted apoptosis of C8‐D1A cells, whereas knockdown of VDAC1 inhibited apoptosis of C8‐D1A cells.
3. Knockdown of NDUFA4 activated ER stress, whereas knockdown of VDAC1 inhibited ER stress.
4. NDUFA4 interacted with VDAC1.

## 1. Introduction

Dandy–Walker malformation (DWM) is a rare congenital brain defect characterized by vermian agenesia, cystic dilation of the fourth ventricle, and enlarged posterior fossa [1, 2]. It represents the most common type of posterior fossa deformity and is usually diagnosed before 1 year of age [3]. In addition to these typical manifestations, DWM is characterized by other abnormalities and malformations of the central nervous system, such as agenesis of the corpus callosum, ectopia, occipital meningocele, visual defects, and epilepsy [4]. DWM patients sometimes exhibit psychotic features, violent and impulsive behavior, or emotional symptoms [5]. However, the pathological mechanism of DWM has not been fully elucidated. DWM is a clinically and genetically heterogeneous condition, and its underlying mechanisms are likely complex. Therefore, it is challenging to explore new anti‐DWM strategies further, and studies focusing on molecular and cellular changes associated with genes implicated in DWM may provide supportive insight, rather than direct causal explanations, for exploring new therapeutic approaches.

NDUFA4, which is encoded by the NDUFA4 gene situated on human Chromosome 7p21.3, plays a crucial role as a component of mitochondrial respiratory chain Complex IV and is integral to mitochondrial energy metabolism [6]. The maintenance of cytochrome C oxidase requires NDUFA4 [7]. Our previous study identified a critical region containing the NDUFA4 gene associated with DWM and limited to 7p21.3 [8]. Additionally, DWM patients have insufficient NDUFA4 gene in the 7p21.3 chromosome region [9]. These genetic findings suggest that NDUFA4 may be a candidate gene associated with DWM, but they do not establish a causal relationship. Based on the function and expression pattern of NDUFA4, it is reasonable to further investigate the cellular effects of NDUFA4 deficiency in neural cells.

Further studies have shown that NDUFA4 could trigger growth factors, inhibit neuronal apoptosis, and promote neuronal growth through the pathway mediated by Bcl‐2 and cytochrome C [10]. By constructing knockout NDUFA4 mice, we found that NDUFA4 affected downstream genes to participate in nerve cell apoptosis by regulating miRNA [11]. These findings indicate that NDUFA4 plays an important role in neuronal survival and apoptosis regulation. However, the mechanism underlying NDUFA4‐mediated regulation of neuronal apoptosis remains to be further investigated.

VDAC1 protein plays a critical role as a regulator of mitochondrial function and serves as the gatekeeper of mitochondria, responsible for cell fate [12]. It maintains mitochondrial function and powers cellular biological activities through energy production [13]. The VDAC1 protein governs essential metabolic and energy functions within cells, such as maintaining Ca^2+^ homeostasis, managing oxidative stress, and modulating mitochondria‐mediated apoptosis [14]. This suggests that regulating VDAC1 is essential not only for mitochondrial metabolic function, but also for cell survival [15]. Notably, VDAC1 emerges as a pivotal convergent node in the pathogenesis of diverse neurodegenerative disorders, including Alzheimer′s disease, Parkinson′s disease, amyotrophic lateral sclerosis (ALS), and Huntington′s disease. In these conditions, VDAC1 is mainly involved in regulating mitochondrial function and neuronal homeostasis. Importantly, previous studies have demonstrated that VDAC1 directly interacts with several misfolded or aggregation‐prone proteins implicated in neurodegenerative diseases, including amyloid‐*β* [16], *α*‐synuclein [17], mutant superoxide dismutase 1 (SOD1) [18], and huntingtin [19], thereby being associated with mitochondrial dysfunction and increased neuronal vulnerability. Shim et al. reported that mitochondrial proteins VDAC1 and NDUFA4 are associated with mitochondrial dysfunction in Alzheimer′s disease pathogenesis [20]. However, the role of NDUFA4–VDAC1 signaling in neurodevelopmental contexts remains unclear.

Based on the above background, we used iTRAQ tandem mass spectroscopy to identify differentially expressed proteins in the cerebellum tissues of 4–6 weeks of wild‐type and knockout mice. We found that VDAC1 expression increased. Accordingly, this study focuses on the cellular mechanisms linking NDUFA4 deficiency to VDAC1‐associated mitochondrial and endoplasmic reticulum dysfunction. Rather than aiming to establish a causal mechanism for DWM, our study seeks to provide functional insight into how altered NDUFA4 expression may affect cellular homeostasis in experimental models, which could be relevant to neurodevelopmental abnormalities observed in DWM.

## 2. Materials and Methods

### 2.1. iTRAQ Tandem Mass Spectrometry

Mice were bred and housed under specific pathogen‐free (SPF) conditions with a 12‐h light/dark cycle, controlled temperature (22^°^C ± 2^°^C), and humidity (50*%* ± 5*%*), and had free access to food and water. In our previous study, we constructed NDUFA4 knockout mice [11] and collected total proteins from cerebellum tissues of wild‐type and knockout mice at 4–6 weeks. After total protein was quantified, trypsin was used to digest the proteins to generate peptide mixtures. The peptide fractions from different groups were separately labeled with iTRAQ reagent tags encoded with different isotopes. Subsequently, the samples were analyzed using a liquid chromatography–tandem mass spectrometry (LC‐MS/MS) system. Volcano plots were generated to visualize differential protein expression. Differentially expressed proteins were analyzed using Gene Ontology (GO) and Kyoto Encyclopedia of Genes and Genomes (KEGG) enrichment analysis in DAVID Version 6.8 (http://david.abcc.ncifcrf.gov). The Top 20 GO terms or the Top 20 KEGG pathways with the most significant enrichment were selected for presentation. Using the online database STRING, protein–protein interactions (PPIs) were predicted, and the PPI network was constructed.

### 2.2. Cell Culture and Treatment

C8‐D1A astrocyte cell (CTCC‐400‐0321, Meisen) was cultured in DMEM (Gibco, Cat. No. 11965092) enriched with 10% fetal bovine serum (Gibco, Cat. No. 10099141C) and 1% penicillin/streptomycin (Gibco, Cat. No. 15140122) at 37°C and 5% CO_2_. Cells were passaged every 2–3 days when reaching 80%–90% confluency. The siRNA targeting NDUFA4 (si‐NDUFA4) and its negative control (si‐NC) were purchased from RiboBio (Guangzhou, China). Cells were seeded into 6‐well plates at a density of 5 × 10^5^ cells/well and transfected with siRNA using Lipofectamine 3000 (Invitrogen, Cat. No. L3000015) according to the manufacturer′s protocol. Cells were divided into si‐NC, si‐NDUFA4‐1, si‐NDUFA4‐2, and si‐NDUFA4‐3 groups (C8‐D1A cells transfected with si‐NC, si‐NDUFA4‐1, si‐NDUFA4‐2, or si‐NDUFA4‐3). After 48 h of transfection, knockdown efficiency was assessed.

The siRNA targeting VDAC1 (si‐VDAC1) and its negative control (si‐NC) were designed and synthesized, and cells were divided into si‐NC, si‐VDAC1‐1, si‐VDAC1‐2, and si‐VDAC1‐3 groups (C8‐D1A cells transfected with si‐NC, si‐VDAC1‐1, si‐VDAC1‐2, or si‐VDAC1‐3). After 48 h of transfection, knockdown efficiency was assessed. C8‐D1A cells were then divided into the following groups: si‐NC group (C8‐D1A cells transfected with si‐NC), si‐NDUFA4 group (C8‐D1A cells transfected with si‐NDUFA4‐3), and si − NDUFA4 + si − VDAC1 group (C8‐D1A cells transfected with si‐NDUFA4‐3 and si‐VDAC1‐3).

The siRNA sequences were as follows: si‐NC‐SS: ACGUGACACGUUCGGAGAA(dT)(dT), si‐NC‐AS: UUCUCCGAACGUGUCACGU(dT)(dT); si‐NDUFA4‐1‐SS: GCUGGGACAGAAAGAACAACC, si‐NDUFA4‐1‐AS: UUGUUCUUUCUGUCCCAGCUG; si‐NDUFA4‐2‐SS: GGAGCAGCACUGUAUGUGAUG, si‐NDUFA4‐2‐AS: UCACAUACAGUGCUGCUCCAG; si‐NDUFA4‐3‐SS: GAAUGUGGACUACAGCAAACU, si‐NDUFA4‐3‐AS: UUUGCUGUAGUCCACAUUCAC; si‐VDAC1‐1‐SS: GCUUCAUACUAAUGUGAAUGA, si‐VDAC1‐1‐AS: AUUCACAUUAGUAUGAAGCUG; si‐VDAC1‐2‐SS: GGCUGACGUUUACAGAGAAGU, si‐VDAC1‐2‐AS: UUCUCUGUAAACGUCAGCCCA; si‐VDAC1‐3‐SS: GGACUGCAGGAAACAGUAACA, si‐VDAC1‐3‐AS: UUACUGUUUCCUGCAGUCCAG.

Next, C8‐D1A cells were divided into the LV‐FLAG‐NDUFA4 group (C8‐D1A cells transfected with LV‐FLAG‐NDUFA4) and the LV‐FLAG‐VDAC1 group (C8‐D1A cells transfected with LV‐FLAG‐VDAC1). Stable plasmids overexpressing NDUFA4 (LV‐FLAG‐NDUFA4, pCMV‐NDUFA4 (mouse)‐3 × FLAG − Neo) and its control plasmid (LV‐NC), as well as stable plasmids overexpressing VDAC1 (LV‐FLAG‐VDAC1, pCMV‐VDAC1 (mouse)‐3 × FLAG − Neo) and its control plasmid (LV‐NC), were purchased from MiaoLing Biology.

### 2.3. Quantitative Real‐Time PCR (qRT‐PCR)

Total RNA was isolated from cells using TRIzol reagent (15596026, Thermo Fisher Scientific). RNA concentrations were measured using a NanoDrop 2000 spectrophotometer (Thermo Fisher Scientific). Reverse transcription was performed using M‐MLV reverse transcriptase (M1701, Promega Corporation). Subsequently, qRT‐PCR was conducted using 2× SYBR Green PCR Mastermix (SR1110, SolarBio Life Sciences). The relative expression levels of NDUFA4 and VDAC1 were calculated by the 2−△△Ct method [21] with *β*‐actin as an internal reference. At least three biological replicates were performed. The primer sequences were as follows: NDUFA4‐F: GGTACTGGAGCAGCACTGTAT, NDUFA4‐R: TTGGGACCCAGTTTGTTCCAT; VDAC1‐F: ACGTGGACTTTGACATCGCT, VDAC1‐R: AAGTTGCTCTGGGTCACTCG; *β*‐actin‐F: GGCTGTATTCCCCTCCATCG; *β*‐actin‐R: CCAGTTGGTAACAATGCCATGT.

### 2.4. Western Blot

Cytoplasmic and mitochondrial proteins were isolated using the Cytoplasmic and Mitochondrial Protein Extraction Kit (C500051‐0050, Sangon Biotech). Western blot analysis was performed to evaluate cytochrome C expression in cytoplasmic proteins (with *β*‐actin as the internal reference) and mitochondrial proteins (with VDAC1 as the internal reference). Total proteins were extracted from cerebellum tissue and C8‐D1A cells using RIPA buffer (R0010, Solarbio Life Science). Protein quantification was performed using the BCA protein assay kit (BL521A, Biosharp). A total of 20 *μ*g of protein per lane was loaded onto SDS‐PAGE gels and transferred to a PVDF membrane. After blocking with 5% BSA, the membranes were incubated with the following primary antibodies: NDUFA4 (A19893, ABCLONAL), VDAC1 (55259‐1‐AP, Proteintech), cytochrome C (A0225, ABCLONAL), caspase‐12 (55238‐1‐AP, Proteintech), caspase‐3 (19677‐1‐AP, Proteintech), Bcl‐2 (26593‐1‐AP, Proteintech), Bax (50599‐2‐Ig, Proteintech), CHOP (A6504, ABCLONAL), ATF4 (A18687, ABCLONAL), PERK (A18196, ABCLONAL), Eif2*α* (A0764, ABCLONAL), and *β*‐actin (BL005B, Biosharp) at 4°C overnight. After washing with TBST, the horseradish peroxidase (HRP)–conjugated goat antirabbit IgG secondary antibody (Proteintech, Cat. No. SA00001‐2, 1:5000 dilution) or HRP‐conjugated goat antimouse IgG secondary antibody (Proteintech, Cat. No. SA00001‐1, 1:5000 dilution) was incubated for 1–2 h. Next, enhanced electrochemiluminescence substrates (32106, Thermo Fisher Scientific) were applied to detect the expression levels of each protein.

### 2.5. Mitochondrial Complex Activity Assay

Following the manufacturer′s instructions, the MitoCheck Complex IV Activity Assay Kit (BC0945, Solarbio) was used to detect respiratory chain Complex IV activity. Briefly, the mitochondrial homogenate was added to the corresponding reaction buffer. The reaction mixture was transferred to a preheated quartz cuvette at 30°C and immediately placed in a UV–Vis spectrophotometer (Shimadzu, UV‐2600). The absorbance was measured at 550 nm to determine Complex IV activity. Mitochondrial complex activity was quantified as nanomoles per minute per milligram of protein.

### 2.6. Cell Counting Kit‐8 (CCK‐8) Assay

C8‐D1A cells were digested into single‐cell suspension with 0.25% trypsin. Following termination of digestion with complete medium, C8‐D1A cells were resuspended in complete medium. C8‐D1A cells were then counted and inoculated into 96‐well plates. Three multiple wells were detected per day for each cell line, and 100 *μ*L of complete medium was added, further cultured at 37°C and 5% CO_2_ in an incubator. After 24, 48 and 96 h, CCK8 reagent (G4103, Sevier) was added. After incubating at 37°C for 2 h, the absorbance at 450 nm was measured using a microplate reader (Thermo Fisher Scientific, Varioskan LUX).

### 2.7. EdU Assay

The proliferation ability of C8‐D1A cells was tested using EdU DNA Proliferation in vitro Detection (C10310, RiboBio), following instructions strictly. C8‐D1A cells were cultured in a medium with 50 *μ*M EdU for 24 h, followed by two washes with PBS for 5 min each. C8‐D1A cells were fixed using 4% paraformaldehyde and 2 mg/mL glycine. Subsequently, they were treated with 1× Apollo reaction solution in the dark for 30 min and then stained with 1× Hoechst33342 reaction solution. Observations were performed immediately after staining.

### 2.8. Flow Cytometry of Cell Apoptosis

An apoptosis detection kit (KGA108, KeyGen) was applied to determine apoptosis of C8‐D1A cells. No EDTA pancreatic enzymes were used to digest and collect the cells. A total of 100‐*μ*L aliquot of the cell suspension was transferred to a tube, adding 5 *μ*L of Annexin V and 5 *μ*L of 7‐AAD. C8‐D1A cells were thoroughly mixed and incubated at room temperature for 15 min, shielded from light. Subsequently, 400 *μ*L of binding buffer was added, and C8‐D1A cells were analyzed by flow cytometer (BD Biosciences, FACSCanto II) within 1 h.

### 2.9. Detection of Ca^2+^ in ER and Mitochondria

Ca^2+^ distribution in mitochondria or ER was evaluated by colocalization. Briefly, Hank′s balanced salt solution was used to prepare a staining solution containing a membrane‐permeable Ca^2+^ fluorescent probe (40703ES50, Yeasen) at a concentration of 5 *μ*M and an ER red fluorescent probe (live cell, 40764ES20, Yeasen) at a concentration of 1 *μ*M. For the detection of Ca^2+^ in mitochondria, Hank′s balanced salt solution was used to prepare a staining solution containing a membrane‐permeable Ca^2+^ fluorescent probe (40703ES50, Yeasen) at a concentration of 5 *μ*M and a mitochondrial red fluorescent probe (40740ES50, Yeasen) at a concentration of 2 *μ*M. After 48 h of culture, cells were washed twice with Hank′s balanced salt solution, and the Ca^2+^ working solution was added. After incubation for 15, 30, or 45 min, red and green fluorescence signals were observed under a laser scanning confocal microscope (Zeiss, LSM 880). The staining solution was then replaced with fresh Hank′s balanced salt solution. DAPI dye at a final concentration of 10 *μ*M was added. After staining for 10 min, Hank′s balanced salt solution was replaced, and images were acquired using a laser scanning confocal microscope.

### 2.10. Transmission Electron Microscopy (TEM)

C8‐D1A cells were collected and fixed at 4°C for 2 h before adding 2.5% neutral glutaraldehyde. Cells were washed with 0.1‐M PBS, fixed with 1% osmic acid (18456, Ted Pella) at 4°C for 1 h and then washed with 0.1‐M PBS. Then, the resin was permeated for 3–4 h after dehydrating using a gradient concentration of ethanol and acetone. Then, it was embedded, solidified, ultrathin sliced, and finally stained with uranium lead. Mitochondria and ER damage were observed with TEM (JEM‐1400 Plus, JEOL Ltd.), and required images were collected.

### 2.11. Detection of ATP

The culture medium was removed. Then, 200 *μ*L of lysis buffer was added to each well of the 6‐well plate for cell lysis. After lysis, the samples were centrifuged at 12,000 × g and 4°C for 5 min. The supernatant was collected and subjected to ATP measurement using an ATP assay kit (S0026, Beyotime).

### 2.12. Immunofluorescence (IF)

When cell confluency reached approximately 80%, C8‐D1A cells were placed on coverslips and seeded into 24‐well plates. IF was performed to observe the colocalization of NDUFA4 and VDAC1 in C8‐D1A cells. Coverslips were fixed with 4% paraformaldehyde and then permeabilized with 0.5% Triton X‐100 at 37°C for 30 min. After rinsing with PBS, cells were blocked with 5% BSA (BS114‐100 g, Biosharp) at 37°C for 30 min, followed by incubation with anti‐NDUFA4 antibody (A19893, ABclonal, 1:200 dilution), anti‐VDAC1 antibody (55259‐1‐AP, Proteintech, 1:200 dilution), and the neuronal marker NeuN (Recombinant Rabbit Monoclonal Antibody [SR45‐07], ET1602‐12, HuaAn Biotechnology, 1:200 dilution) overnight at 4°C. Secondary antibodies (FITC‐conjugated goat antirabbit IgG, Proteintech, SA00003‐2, 1:500; TRITC‐conjugated goat antimouse IgG, Proteintech, SA00003‐3, 1:500) were applied for 90 min at 37°C. Nuclei were stained with DAPI (Solarbio, C0060, 1:1000) for 10 min at 37°C. Coverslips were mounted with glycerol‐based mounting medium and examined under a fluorescence microscope (Olympus, BX53).

For mouse cerebellar tissue, sections were baked at 60°C for 12 h and dewaxed to water, rehydrated through graded ethanol series (100%, 100%, 95%, 85%, and 75%; 5 min each), and soaked in distilled water for 5 min. Antigen retrieval was performed by heat treatment, followed by incubation with 3% H_2_O_2_ for 10 min to block endogenous peroxidase activity. Sections were blocked with 5% BSA and incubated overnight at 4°C with primary antibodies against NDUFA4, VDAC1, and NeuN. Secondary antibodies were applied for 90 min at 37°C, and nuclei were stained with DAPI for 10 min at 37°C. Sections were mounted with antifade medium (G1401, Servicebio) and visualized under a fluorescence microscope.

### 2.13. Coimmunoprecipitation (Co‐IP)

To detect whether there was an interaction between NDUFA4 and VDAC1, a Co‐IP assay was performed using an anti‐FLAG antibody. Cells were harvested for protein extraction. Subsequently, extracts with equal protein amounts were subjected to immunoprecipitation in lysis buffer containing NDUFA4 or VDAC1 antibodies at 4°C overnight. Protein A/G agarose beads were then added to the immunoprecipitation mixture for 2 h and washed three times. The immunoprecipitated complexes were resuspended in SDS‐PAGE sample buffer, boiled, separated by SDS‐PAGE, and transferred to a PVDF membrane. The membranes were incubated with NDUFA4 and VDAC1 primary antibodies, followed by incubation with the corresponding secondary antibodies. Next, enhanced electrochemiluminescence substrates (32106, Thermo Fisher Scientific) were applied to detect the protein signals.

### 2.14. Statistical Analysis

Statistical analysis was conducted using GraphPad Prism 8.0 software. Data are presented as mean ± standard deviation. Student′s *t*‐test was used for comparisons between two groups, whereas one‐way analysis of variance (ANOVA) was applied for comparisons among multiple groups. A *p* value < 0.05 was considered statistically significant. All experiments were independently performed at least three times.

## 3. Results

### 3.1. iTRAQ Tandem Mass Spectrometry Analysis of Protein Expression Changes in NDUFA4 Knockout Mice

**Figure 1:**
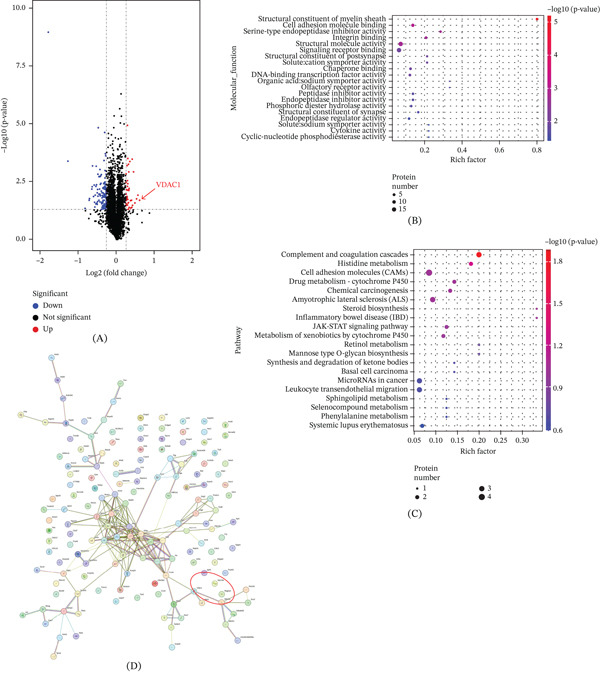
iTRAQ tandem mass spectrometry analysis of protein expression changes in NDUFA4 knockout mice. (A) Volcano plot analysis of differentially expressed proteins. (B) GO analysis of the molecular functions of differentially expressed proteins. (C) KEGG pathway analysis of differentially expressed proteins. (D) PPI analysis of predicted interactions among differentially expressed proteins.

In our previous study, we constructed NDUFA4 knockout mice [11], collected cerebellum tissues of wild‐type and knockout mice at 4–6 weeks of age to extract total protein, and analyzed protein expression changes associated with NDUFA4 knockout by iTRAQ tandem mass spectrometry. First, the volcano map showed a total of 158 differentially expressed proteins. Among them, 116 showed decreased expression, and 42 showed increased expression (Figure 1A). Next, GO analysis of the differentially expressed proteins showed enrichment mainly in structural molecule activity, receptor binding, and cell adhesion molecule (CAM) binding (Figure 1B). KEGG pathway analysis indicated that these proteins were mainly enriched in CAMs, ALS, complement and coagulation cascades, microRNAs in cancer, and leukocyte transendothelial migration pathways (Figure 1C). PPI analysis identified VDAC1 as an upregulated protein showing a predicted interaction with NDUFA4 (Figure 1D).

### 3.2. Effects of NDUFA4 Knockout on VDAC1 Expression and Mitochondrial‐Related Cellular Changes

**Figure 2:**
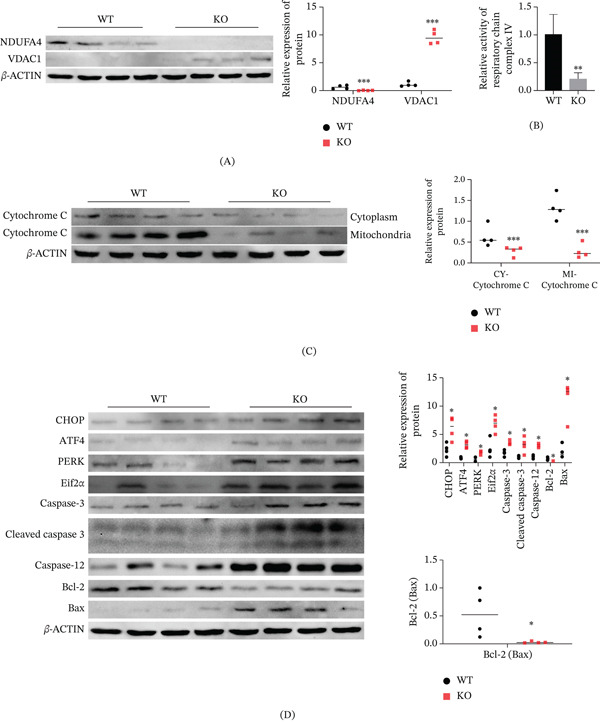
Effects of NDUFA4 knockout on VDAC1 expression and mitochondrial‐related cellular changes. Cerebellum tissues from four pairs of wild‐type and NDUFA4 knockout mice were collected. (A) NDUFA4 and VDAC1 expression detected by Western blot. (B) Activity of ETC Complex IV measured using the MitoCheck Complex IV Activity Assay Kit. (C) Western blot analysis of cytochrome C expression in mitochondrial and cytoplasmic fractions. (D) Western blot analysis of apoptosis‐related proteins (caspase‐3, cleaved caspase‐3, caspase‐12, Bax, and Bcl‐2) and ER stress–related proteins (CHOP, ATF4, PERK, and Eif2*α*). (*n* = 4).  ^∗^ *p* < 0.05,  ^∗∗∗^ *p* < 0.001,  ^∗∗∗∗^ *p* < 0.0001.

VDAC1 expression was elevated, whereas NDUFA4 expression was reduced in the cerebellum tissues of NDUFA4 knockout mice (Figure 2A). The activity of ETC Complex IV in mouse cerebellum tissue decreased after NDUFA4 knockout (Figure 2B), which is consistent with the reported role of NDUFA4 as a component of ETC Complex IV. Moreover, after NDUFA4 knockout, cytochrome C expression in both mitochondrial and cytoplasmic fractions of mouse cerebellum tissue was decreased (Figure 2C). Cytochrome C is involved in mitochondrial electron transport and apoptosis‐related processes, and altered cytochrome C distribution is commonly associated with mitochondrial dysfunction. In addition, NDUFA4 knockout led to increased expression of ER stress–related proteins (CHOP, ATF4, PERK, and Eif2*α*) and apoptosis‐related proteins (caspase‐3, cleaved caspase‐3, caspase‐12, and Bax), whereas Bcl‐2 expression was decreased (Figure 2D). These results indicate that NDUFA4 deficiency is associated with the activation of ER stress responses and apoptotic signaling in cerebellar tissue.

### 3.3. Effects of NDUFA4 Knockdown and Subsequent VDAC1 Knockdown on Cellular Activity

**Figure 3:**
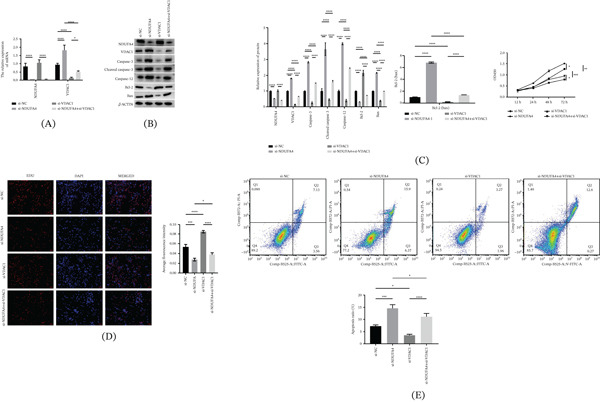
Effects of NDUFA4 knockdown and subsequent VDAC1 knockdown on cellular activity. After transfection with si‐NDUFA4 and si‐VDAC1, (A) qRT‐PCR analysis of NDUFA4 and VDAC1 mRNA expression. (B) Western blot analysis of NDUFA4, VDAC1, and apoptosis‐related proteins (caspase‐3, cleaved caspase‐3, caspase‐12, Bax, and Bcl‐2). (C, D) CCK‐8 assay and EdU staining used to assess C8‐D1A cell proliferation. (E) Apoptosis of C8‐D1A cells measured by flow cytometry. (*n* = 3).  ^∗^ *p* < 0.05,  ^∗∗^ *p* < 0.01,  ^∗∗∗^ *p* < 0.001,  ^∗∗∗∗^ *p* < 0.0001.

First, NDUFA4 and VDAC1 were knocked down using siRNA. Compared with the si‐NC group, the mRNA and protein expression levels of NDUFA4 in the si‐NDUFA4‐1, si‐NDUFA4‐2, and si‐NDUFA4‐3 groups were decreased (Figure S1A,B). Among these, si‐NDUFA4‐3 showed the highest knockdown efficiency and was therefore used in subsequent experiments. Similarly, compared with the si‐NC group, the mRNA and protein expression levels of VDAC1 in the si‐VDAC1‐1, si‐VDAC1‐2, and si‐VDAC1‐3 groups were decreased (Figure S1C,D). Si‐VDAC1‐3 exhibited the highest knockdown efficiency and was selected for further experiments. Next, combined knockdown experiments were performed. In the si‐NDUFA4 group, NDUFA4 mRNA and protein levels were decreased compared with those in the si‐NC group, whereas VDAC1 mRNA and protein levels were increased. Subsequent knockdown of VDAC1 resulted in reduced VDAC1 mRNA and protein levels (Figure 3A,B). Moreover, in the si‐NDUFA4 group, the expression levels of apoptosis‐related proteins (caspase‐3, cleaved caspase‐3, caspase‐12, and Bax) were increased, whereas Bcl‐2 expression was decreased compared with those in the si‐NC group. Additional knockdown of VDAC1 attenuated these changes, leading to decreased expression of caspase‐3, cleaved caspase‐3, caspase‐12, and Bax and increased Bcl‐2 expression (Figure 3B). Cell functional assays showed that C8‐D1A cell proliferation was reduced and apoptosis was increased in the si‐NDUFA4 group compared with the si‐NC group. Subsequent knockdown of VDAC1 partially reversed these changes, with increased cell proliferation and reduced apoptosis (Figure 3C–E). In addition, data from the si‐VDAC1 group (C8‐D1A cells transfected with si‐VDAC1‐3 alone) were included in Figures 3A–C and E to demonstrate the independent effects of VDAC1 knockdown.

### 3.4. Effects of NDUFA4 Knockdown and Subsequent VDAC1 Knockdown on Mitochondrial and ER‐Associated Changes

Compared with the si‐NC group, the expression levels of ER stress–related proteins (CHOP, ATF4, PERK, and Eif2*α*) were increased in the si‐NDUFA4 group. Subsequent knockdown of VDAC1 attenuated the upregulation of CHOP, ATF4, PERK, and Eif2*α* (Figure 4A). In addition, cytochrome C expression in both mitochondrial and cytoplasmic fractions was decreased in the si‐NDUFA4 group compared with the si‐NC group. Following additional VDAC1 knockdown, cytochrome C expression showed a relative increase (Figure 4B). Moreover, NDUFA4 knockdown was associated with decreased ETC Complex IV activity, whereas additional VDAC1 knockdown partially restored complex IV activity (Figure 4C). Fluorescence probe analysis showed that NDUFA4 knockdown was associated with altered Ca^2+^ distribution, characterized by increased mitochondrial Ca^2+^ signals and reduced ER‐associated Ca^2+^ signals. Additional knockdown of VDAC1 partially reversed these Ca^2+^ distribution patterns (Figure 4D,E). TEM revealed that NDUFA4 knockdown was associated with mitochondrial structural alterations, including reduced mitochondrial size, disrupted mitochondrial membranes, and decreased or absent cristae, as well as ER dilation. Following VDAC1 knockdown, these ultrastructural alterations were partially alleviated (Figure 4F). ATP measurements were performed to further assess mitochondrial function. ATP levels were decreased after NDUFA4 knockdown, and additional VDAC1 knockdown partially reversed this decrease (Figure 4G).

Figure 4Effects of NDUFA4 knockdown and subsequent VDAC1 knockdown on mitochondrial and ER‐associated changes. After transfection with si‐NDUFA4 and si‐VDAC1. (A) ER stress–related protein expression (CHOP, ATF4, PERK, and Eif2*α*) detected by Western blot. (B) Western blot analysis of cytochrome C expression in mitochondrial and cytoplasmic fractions. (C) Activity of ETC Complex IV measured using the MitoCheck Complex IV Activity Assay Kit. (D) Analysis of mitochondrial Ca^2+^ distribution using fluorescence probes. (E) Analysis of ER‐associated Ca^2+^ distribution using fluorescence probes. (F) TEM observation of mitochondrial and ER ultrastructural alterations. (G) Measurement of ATP levels. (*n* = 3).  ^∗^ *p* < 0.05,  ^∗∗^ *p* < 0.01,  ^∗∗∗^ *p* < 0.001,  ^∗∗∗∗^ *p* < 0.0001.

**Figure:**
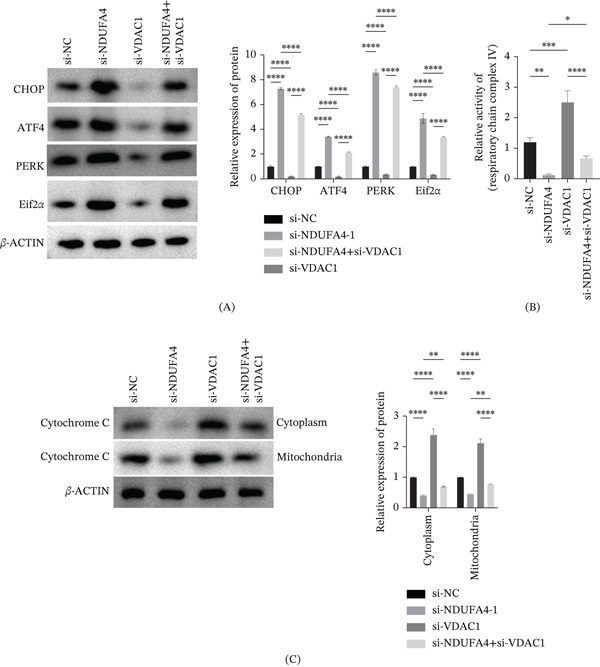
(A)

**Figure:**
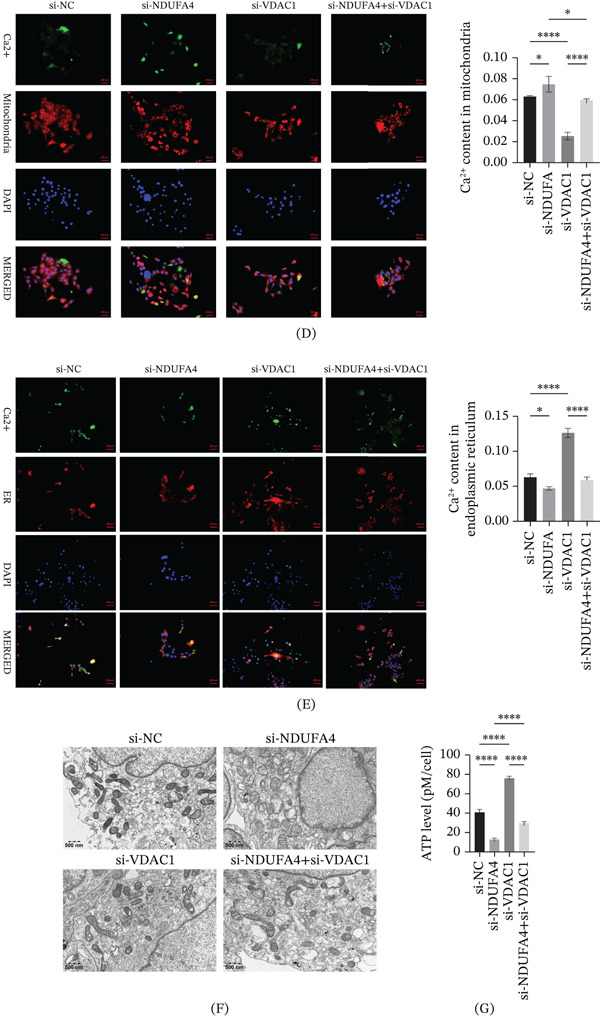
(B)

### 3.5. Evidence for Interaction Between NDUFA4 and VDAC1

**Figure 5:**
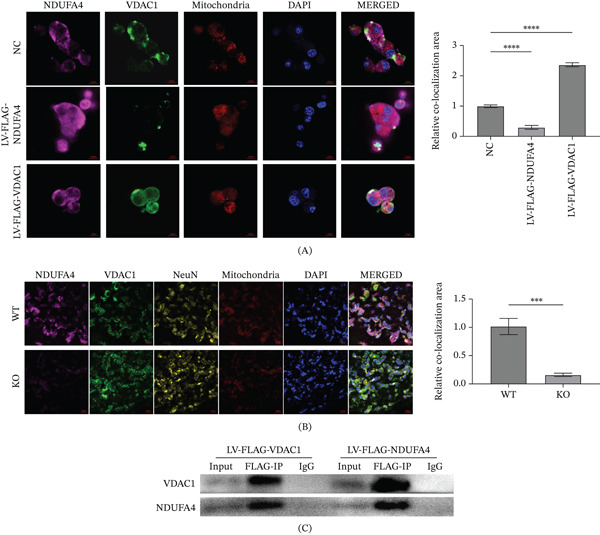
Evidence for an association between NDUFA4 and VDAC1. (A) Colocalization of NDUFA4 and VDAC1 in C8‐D1A cells detected by IF. (B) IF staining of WT and NDUFA4 knockout mouse cerebellar tissue showing colocalization of NDUFA4 and VDAC1, with signals also observed in NeuN‐positive cells. (C) Co‐IP analysis showing NDUFA4 detected in VDAC1‐containing immunoprecipitates. (*n* = 3).  ^∗∗∗∗^ *p* < 0.0001.

The results showed that NDUFA4 and VDAC1 were colocalized in C8‐D1A cells (Figure 5A). In addition, NDUFA4 and VDAC1 were also colocalized in mouse cerebellar tissue, with signals observed in NeuN‐positive cells (Figure 5B). NDUFA4 is localized to the inner mitochondrial membrane, whereas VDAC1 is localized to the outer mitochondrial membrane. Colocalization signals were observed in mitochondrial regions, which is consistent with previous reports showing that proteins residing in the inner and outer mitochondrial membranes can spatially associate at mitochondrial contact sites, where the two membranes are closely apposed and functionally coupled [22, 23]. Furthermore, co‐IP assays demonstrated that NDUFA4 was detected in VDAC1‐containing immunoprecipitates, indicating an association between NDUFA4 and VDAC1 (Figure 5C).

## 4. Discussion

DWM is a rare abnormality in the brain that arises during the development of the cerebellum and fourth ventricle in the embryonic stage [24]. DWM is linked to various neurodevelopmental issues, including cognitive, motor, and behavioral disorders that go beyond just microcephaly. It is crucial to acknowledge that the underlying causes of these symptoms are still not fully understood [21]. Against this background, elucidating cellular and molecular alterations associated with candidate genes may provide mechanistic clues, even in the absence of direct causal evidence. Hence, there is an imminent requirement to investigate novel therapies and the pathogenesis of DWM to gain fresh perspectives for identifying innovative therapeutic targets. Given these uncertainties, studies focusing on cellular changes related to candidate genes may provide supportive insight, but they do not establish causality. In line with this rationale, the present study provides evidence that NDUFA4 deficiency is associated with VDAC1 upregulation, mitochondrial‐related alterations, ER stress responses, and apoptotic signaling in experimental models. These findings may be relevant to neurodevelopmental abnormalities observed in DWM, but they do not demonstrate that NDUFA4 changes directly cause DWM. Rather, our data provide a cellular framework suggesting that altered NDUFA4 expression may disturb cellular homeostasis in experimental models. To our knowledge, this is the first study to examine the NDUFA4–VDAC1 association in the context of DWM‐related neurodevelopmental research.

Based on our previous study [11], we identified many NDUFA4‐regulated differential target proteins, which are mainly enriched in structural molecule activity, receptor binding, and CAM binding. These enrichment patterns suggest that NDUFA4 may affect multiple cellular processes through interconnected pathways rather than through a single downstream effector. Besides, KEGG analysis revealed differentially expressed proteins involved in CAMs, ALS, complement and coagulation cascades, microRNAs in cancer, and leukocyte transendothelial migration. Although these pathways span diverse biological processes, their convergence on neural structure‐related and stress‐related responses provides a rationale for further mechanistic exploration. These observations require further validation in independent cohorts and experimental settings. Moreover, PPI analysis revealed that the protein VDAC1 with elevated expression showed a predicted interaction with NDUFA4, thereby highlighting VDAC1 as a potential molecular node linking NDUFA4 deficiency to mitochondrial dysfunction in DWM‐related models.

NDUFA4 is a respiratory chain–oxidative phosphorylation pathway component highly expressed in tumor tissues [25]. Cytochrome C oxidase is an enzyme positioned after the electron transport chain, facilitating the transfer of electrons from cytochrome C to oxygen [26]. NDUFA4 may be an assembly factor of cytochrome C oxidase or supercomplex (respiratory body) in mitochondria of growing cells and cancer tissues [27]. Consistent with this functional role, previous studies have shown that perturbation of NDUFA4 expression is associated with mitochondrial dysfunction in different pathological contexts. Zhu et al. reported that miR‐147 inhibited NDUFA4 and induced mitochondrial dysfunction and tubule injury in cold storage kidney transplantation [28]. Chen et al. revealed that miR‐210‐3p enhanced cardiomyocyte apoptosis and mitochondrial dysfunction in sepsis‐induced myocardial dysfunction by targeting the NDUFA4 gene [29]. In ALS, REEP1 improved mitochondrial function by interacting with NDUFA4 and protected the motor function of SOD1G93A mice [30]. Our previous studies have shown that NDUFA4 could significantly enhance neuronal activity and inhibit neuronal apoptosis [10]. Building on these observations, we found that NDUFA4 deficiency in cerebellar tissue from knockout mice was associated with VDAC1 upregulation, reduced ETC Complex IV activity and cytochrome C expression, ER stress activation, and apoptosis‐related changes. Taken together, these findings support the involvement of NDUFA4 in mitochondrial homeostasis, ER stress responses, and apoptosis‐related signaling in cerebellar tissue. However, these data should be interpreted as model‐based cellular evidence rather than direct proof of a disease mechanism in DWM.

VDAC1 is an essential substrate of Parkin [31]. VDAC1 plays a crucial role in mitochondria‐mediated apoptosis by releasing apoptotic proteins in the intermembrane space and interacting with proapoptotic and antiapoptotic proteins. Consequently, VDAC1 is a promising target for apoptosis regulation [32]. In addition, through the transport of Ca^2+^, VDAC1 serves a vital function in controlling mitochondrial Ca^2+^ balance, oxidative phosphorylation, and the exchange of Ca^2+^ between mitochondria, cytoplasm, and the ER [33]. Tiwari et al. found that UBA52 regulated VDAC1‐mediated mitochondrial dysfunction and dopaminergic neuronal death [34]. Furthermore, VDAC1 could regulate the loss of neuronal cells after retinal injury through a mitochondrial‐independent pathway [35]. Inhibition of VDAC1 could protect HT22 cells from oxidative stress and mitochondrial fracture induced by glutamate [36]. These studies indicate that VDAC1 is closely related to mitochondrial stress responses and neuronal survival across different experimental contexts. In our models, VDAC1 upregulation was associated with reduced Complex IV activity and changes in Ca^2+^ distribution, which is consistent with a role of VDAC1 in mitochondrial regulation.

The mechanistic link between NDUFA4 and VDAC1 expression requires further investigation. At present, the regulatory relationship between NDUFA4 and VDAC1 remains unclear. One possibility is that NDUFA4 deficiency triggers mitochondrial stress signals that secondarily affect VDAC1 expression. This hypothesis needs further experimental testing. Regarding the physical interaction between NDUFA4 and VDAC1, our IF and co‐IP data support an association between the two proteins in our experimental systems. Previous studies have shown that proteins residing in the inner and outer mitochondrial membranes can spatially associate at mitochondrial contact sites, where the two membranes are closely apposed and functionally coupled [22]. The biological significance of the NDUFA4–VDAC1 association remains to be clarified, including whether it influences Ca^2+^ handling or metabolite exchange. Notably, VDAC1 has been reported to participate in Ca^2+^ transfer between mitochondria and the endoplasmic reticulum and to contribute to mitochondrial Ca^2+^ homeostasis and stress responses [37]. In our experimental models, VDAC1 upregulation was associated with altered Ca^2+^ distribution between mitochondria and the ER, which is consistent with these previously described roles of VDAC1 in mitochondrial–ER communication. The upregulation of VDAC1 in NDUFA4 knockout models may be associated with changes in multiple mitochondrial‐related processes beyond apoptosis. In our study, VDAC1 upregulation was associated with reduced complex IV activity and altered Ca^2+^ distribution in mitochondria and the ER. These findings are consistent with mitochondrial and ER stress responses observed after NDUFA4 deficiency. However, the current data do not define a direct causal chain from these cellular changes to DWM.

In this study, through further in vitro cell experiments, we found that interfering with NDUFA4 promoted apoptosis of C8‐D1A cells and activated ER stress, while interfering with VDAC1 inhibited apoptosis of C8‐D1A cells and inhibited ER stress. These observations suggest that VDAC1 may modulate apoptosis‐ and ER stress–related changes induced by NDUFA4 deficiency in C8‐D1A cells. To our knowledge, this is the first report describing the NDUFA4–VDAC1 association in relation to apoptosis‐ and ER stress–related readouts in these models. Finally, IF staining showed colocalization of NDUFA4 and VDAC1 in cultured cells and in mouse cerebellar tissue, with signals also observed in NeuN‐positive cells. The co‐IP experiment further supported an association between NDUFA4 and VDAC1. Overall, our data suggest that NDUFA4 deficiency is linked to VDAC1‐associated mitochondrial and ER alterations in experimental systems. These findings may be relevant to DWM, but they do not confirm that NDUFA4 and VDAC1 directly drive DWM.

An additional limitation of this study is that the key mechanistic experiments were performed in the C8‐D1A astrocyte cell line rather than in neuronal cells. We selected C8‐D1A cells because astrocytes are important for the neural microenvironment and this cell line is relatively stable for in vitro culture and transfection [38, 39]. In addition, mitochondrial dysfunction and ER stress in astrocytes may indirectly influence neuronal survival and function [40]. However, this experimental design does not allow us to determine whether the same NDUFA4–VDAC1 mechanism is also present in neurons. Further studies in more relevant neuronal models, such as primary cerebellar neurons or iPSC‐derived neurons, are needed to determine whether similar changes are also present in neurons.

This study also has other limitations. The independent effects of VDAC1 knockdown on C8‐D1A cell proliferation, apoptosis, and ER stress should be further characterized. In addition, previous studies have shown that VDAC1 overexpression induces VDAC1 oligomerization [41, 42], which is related to mitochondria‐mediated apoptosis [43]. Therefore, the role of VDAC1 oligomerization warrants further study and will be examined in future work.

## 5. Conclusion

Our results suggest that NDUFA4 deletion is associated with VDAC1 upregulation and mitochondrial‐ and ER‐related alterations, together with apoptosis‐related changes in our experimental models. These findings may offer insight into cellular processes that could be relevant to DWM. However, further studies in more relevant neuronal models are needed to clarify cell‐type specificity and to better assess how these findings relate to DWM.

**Nomenclature**
DWM: Dandy–Walker malformation
ER: endoplasmic reticulum
NDUFA4: NADH Dehydrogenase 1 alpha Subcomplex 4
VDAC1: voltage‐dependent anion channel 1
CAMs: cell adhesion molecules
ALS: amyotrophic lateral sclerosis
GO: Gene Ontology
KEGG: Kyoto Encyclopedia of Genes and Genomes
PPI: protein–protein interaction
qRT‐PCR: quantitative real‐time PCR
CCK‐8: Cell Counting Kit‐8
TEM: transmission electron microscopy
IF: Immunofluorescence
Co‐IP: coimmunoprecipitation
ANOVA: analysis of variance

## Author Contributions

Methodology: Lushan Li and Can Liao; data curation, formal analysis, and investigation: Fang Fu, Ru Li, Hang Zhou, and Ruibing Huang; resources and visualization: Jianqin Lu, Fei Guo, Huanyi Chen, Tingying Lei, and Jin Han; validation: Li Zhen, Min Pan, and Dongzhi Li; writing—original draft: Lushan Li; writing—review and editing: Lushan Li, Fang Fu, Ru Li, Hang Zhou, Ruibing Huang, Jianqin Lu, Fei Guo, Huanyi Chen, Tingying Lei, Jin Han, Li Zhen, Min Pan, Dongzhi Li, and Can Liao.

## Funding

This research was supported by the subproject of the National Key R&D Program (Grant Number 2021YFC2701002), the National Natural Science Foundation of China (Grant Numbers 82101796 and 82201884), the Guangdong Basic and Applied Basic Research Foundation (Grant Number 2023A1515010115), Guangzhou Science and Technology Project (Grant Numbers 202201020643, 202201010879, 20221A011029, 202201020604, and 202102020061), and the Research Foundation of Guangzhou Women and Children′s Medical Center for Clinical Doctors (Grant Number 2020BS030).

## Ethics Statement

All experimental procedures using mice were approved by the Experimental Animal Care and Ethics Committee of the Forevergen Medical Laboratory Animal Center, Guangzhou, China (Approval No. IACUC‐G16051).

## Conflicts of Interest

The authors declare no conflicts of interest.

## Supporting information


**Supporting Information** Additional supporting information can be found online in the Supporting Information section. Figure S1 Synthesis of siRNA interfering with NDUFA4 or VDAC1 and verification of interference effects. (A, B) qRT‐PCR and Western blot measurement of NDUFA4 mRNA and protein expression after interference with NDUFA4. (C, D) qRT‐PCR and Western blot were utilized to assess VDAC1 mRNA and protein levels after interference with VDAC1.  ^∗^ *p* < 0.05,  ^∗∗^ *p* < 0.01.

## Data Availability

All data generated or analyzed during this study are included in this published article.
