# Supplementary figures and images for: NDUFA4 Deletion Upregulates VDAC1 to Promote Mitochondrial Damage, Endoplasmic Reticulum Expansion, and Neuronal Apoptosis
Source: Hum Mutat. 2026 May 21;2026:8889386. doi: 10.1155/humu/8889386 (PMC13191783; doi:10.1155/humu/8889386)

A

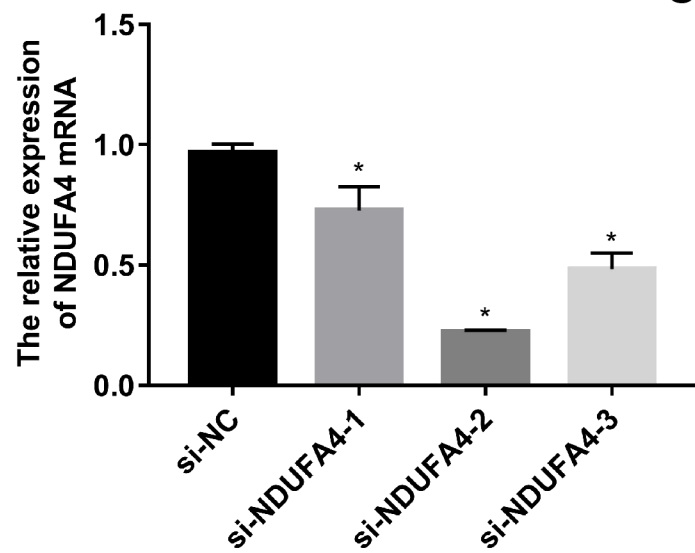

C

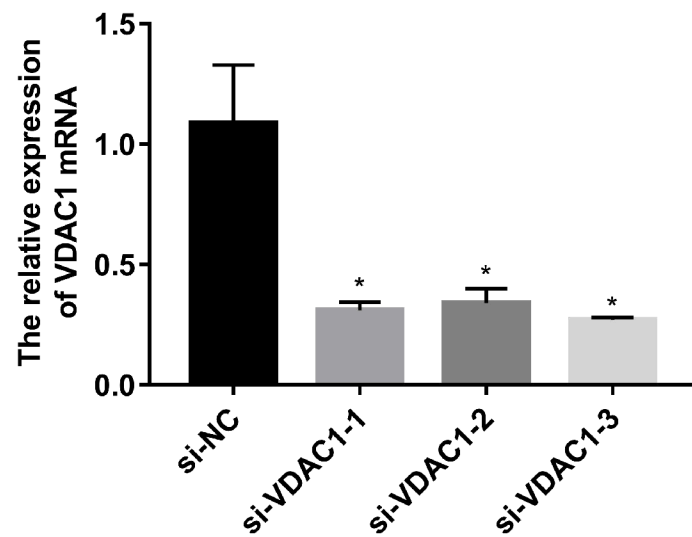

B

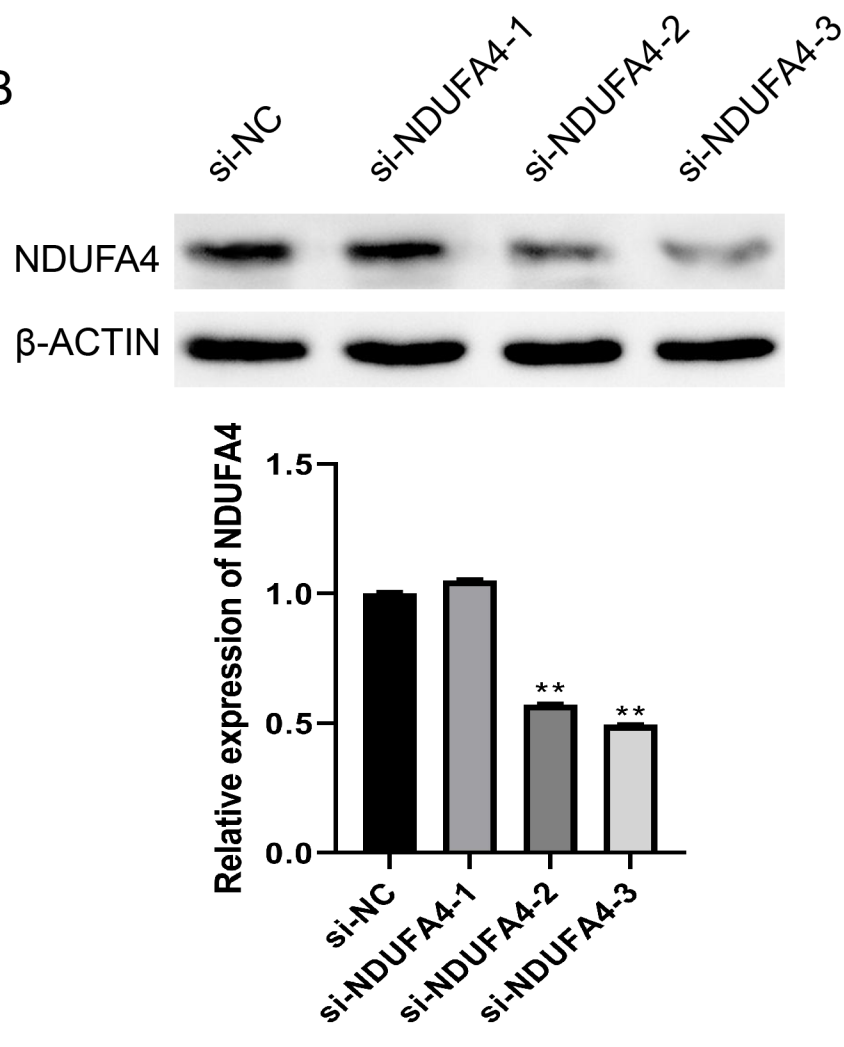

D

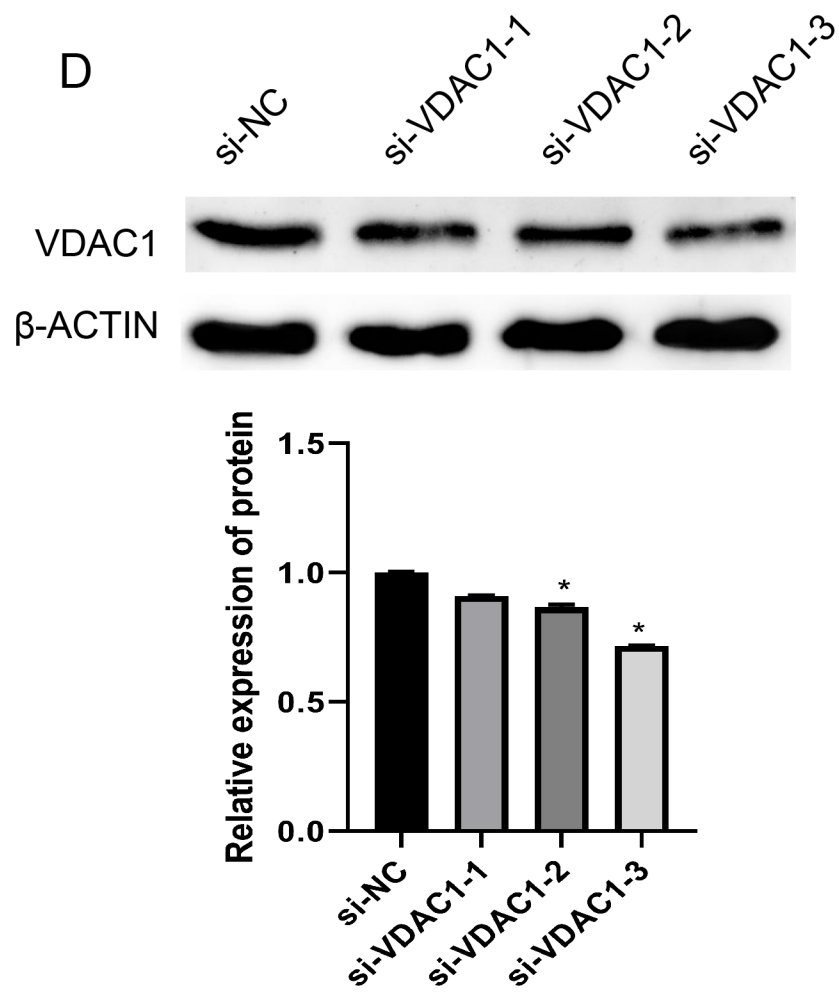

Supplement: Supplementary file 1 — Supporting Information Additional supporting information can be found online in the Supporting Information section. Figure S1 Synthesis of siRNA interfering with NDUFA4 or VDAC1 and verification of interference effects. (A, B) qRT‐PCR and Western blot measurement of NDUFA4 mRNA and protein expression after interference with NDUFA4. (C, D) qRT‐PCR and Western blot were utilized to assess VDAC1 mRNA and protein levels after interference with VDAC1. ∗ p < 0.05, ∗∗ p < 0.01. [file HUMU-2026-8889386-s001.pdf]
